# Supplementary material for: Adding chemically selective subtraction to multi-material 3D additive manufacturing
Source: Nat Commun. 2018 Jul 17;9:2788. doi: 10.1038/s41467-018-05234-0 (PMC6050325; doi:10.1038/s41467-018-05234-0)
Supplement: Supplementary file 1 — Supplementary Information [file 41467_2018_5234_MOESM1_ESM.pdf]

Supplementary Information

Adding Chemically Selective Subtraction to Multi-Material 3D Additive Manufacturing

David Gräfe, Andreas Wickberg, Markus Zieger, Martin Wegener, Eva Blasco,

Christopher Barner-Kowollik

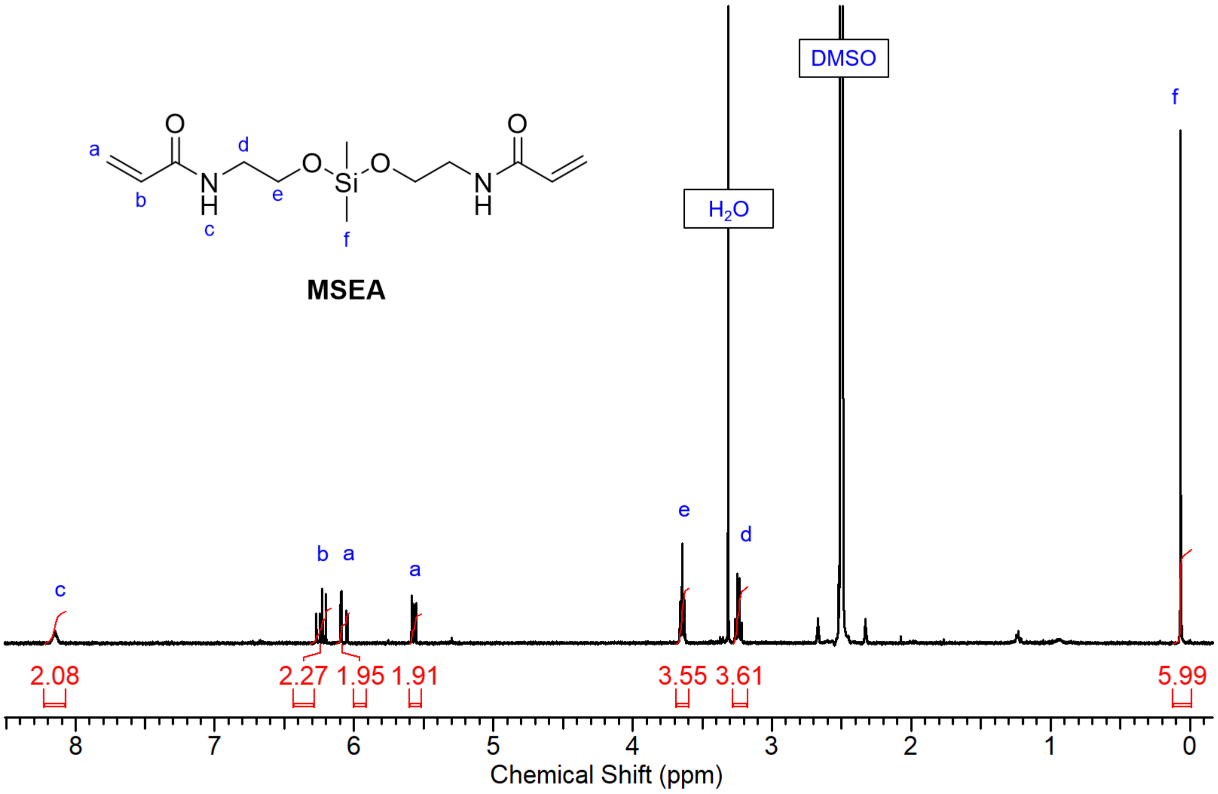

Supplementary Figure 1. ^{1}H NMR spectrum of MSEA in DMSO-d_6.

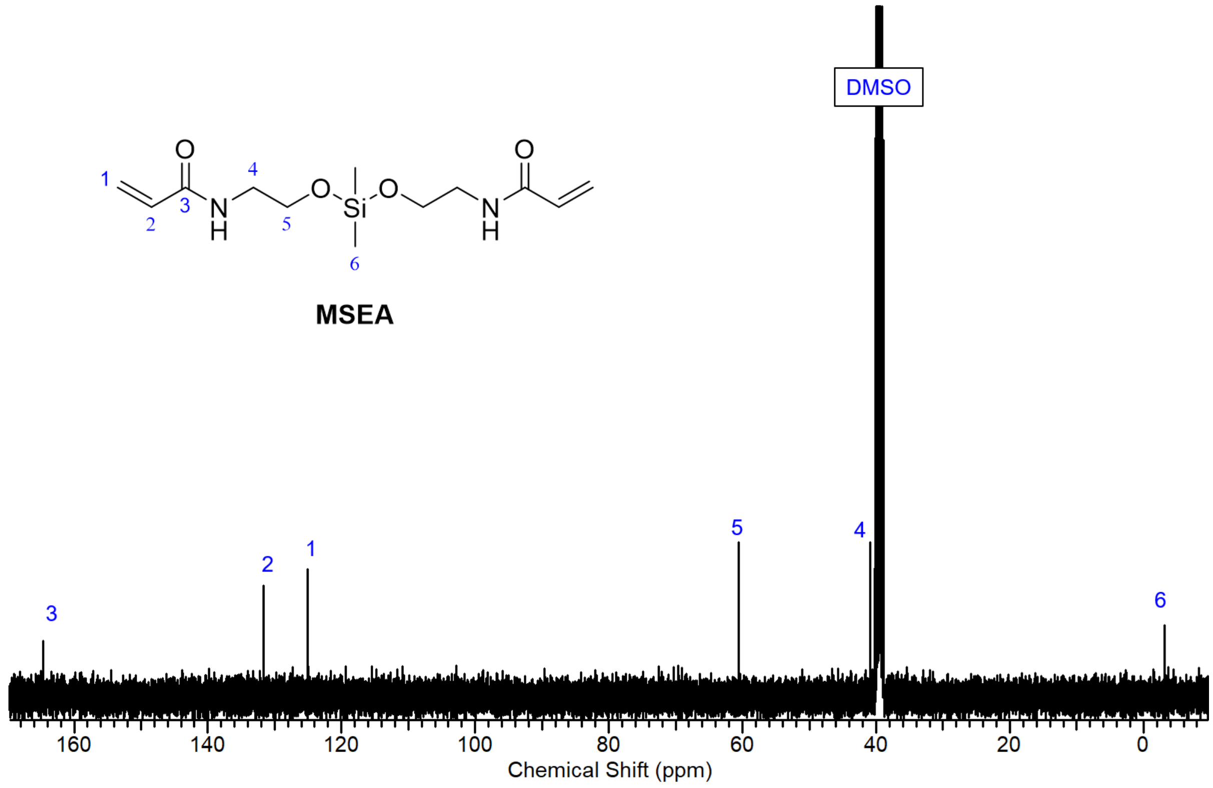

Supplementary Figure 2. ^{13}C NMR spectrum of MSEA in DMSO-d_6.

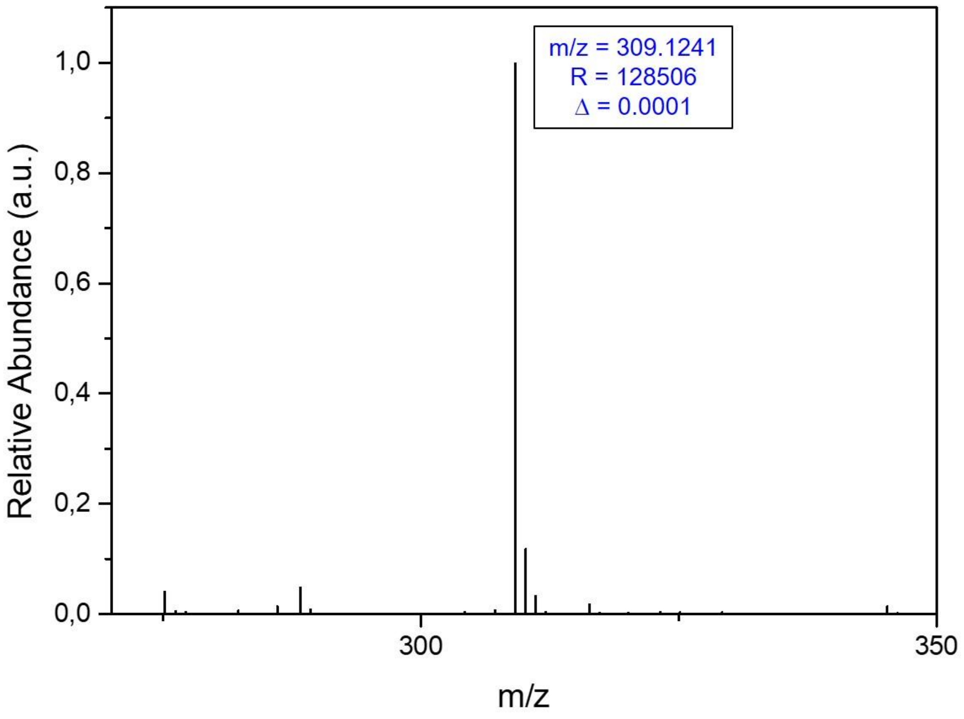

Supplementary Figure 3. ESI MS spectrum of MSEA in DCM / MeOH solution (3 : 1) doped with 0.1 mM sodium trifluoroacetate.

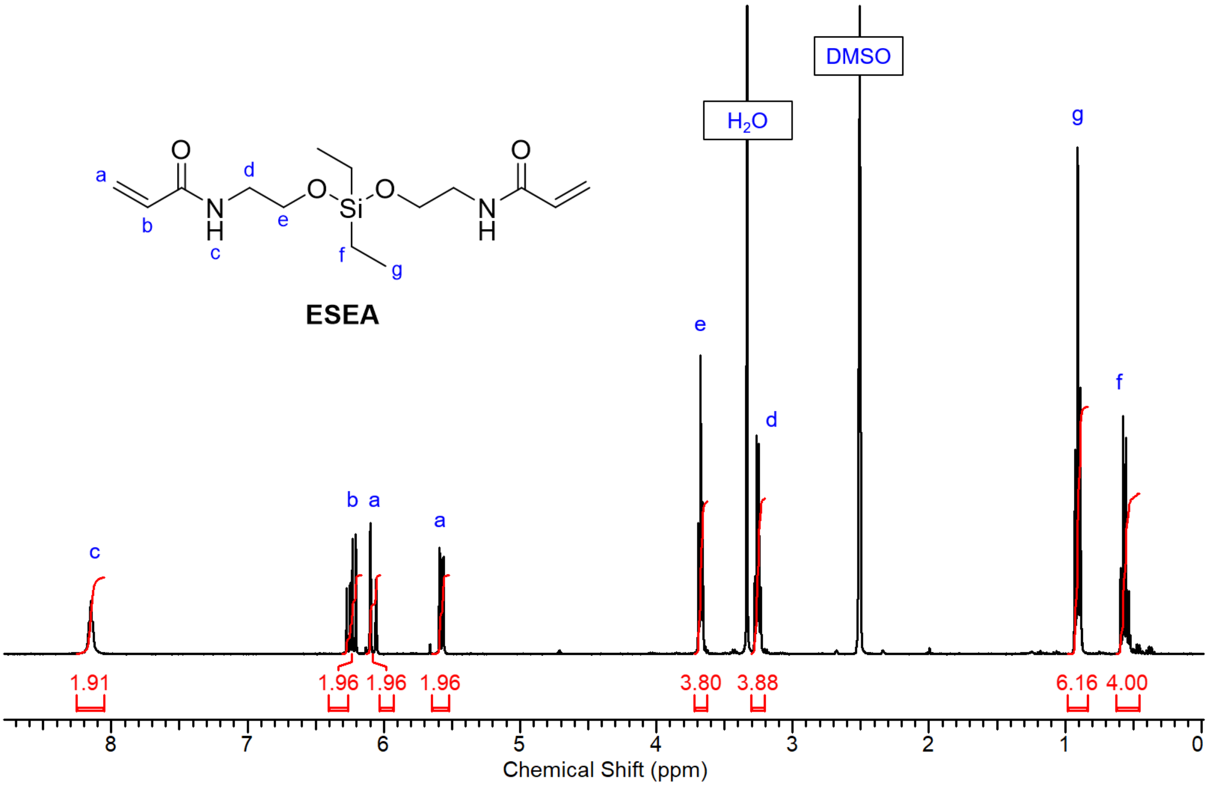

Supplementary Figure 4. ^{1}H NMR spectrum of ESEA in DMSO-d_6.

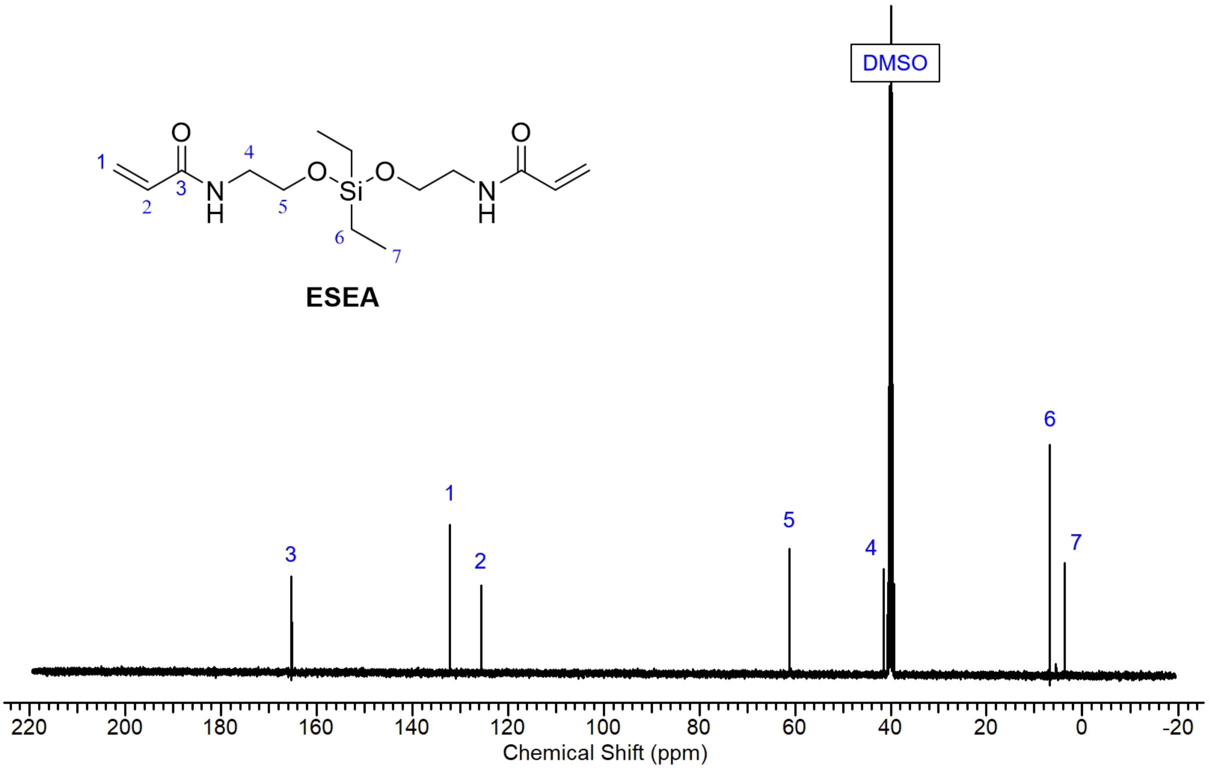

Supplementary Figure 5. ^{13}C NMR spectrum of ESEA in DMSO-d_6.

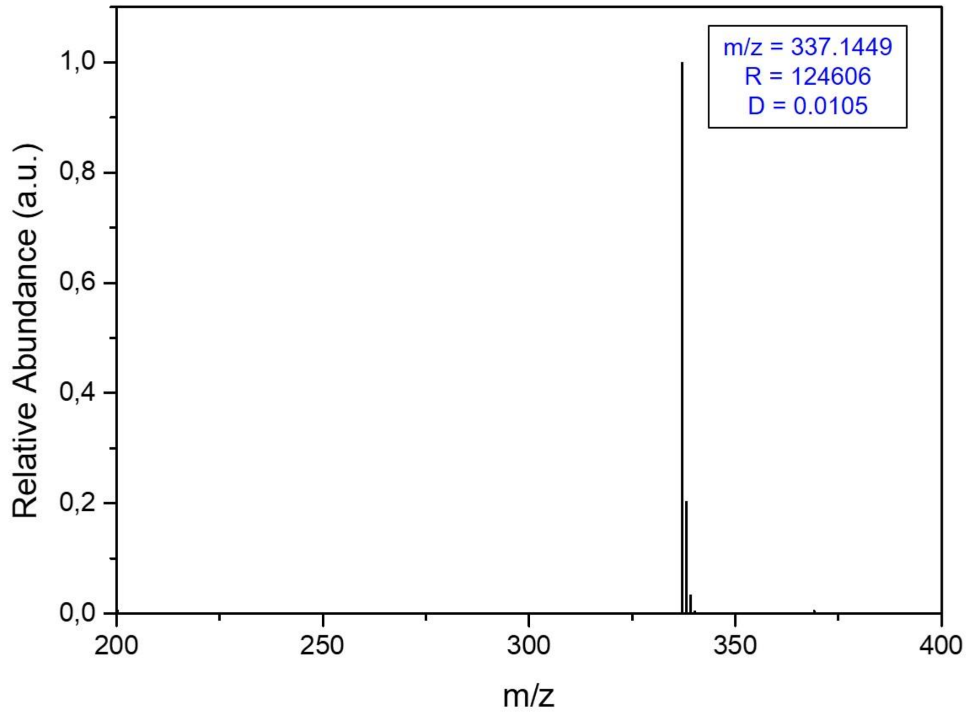

Supplementary Figure 6. ESI MS spectrum of ESEA in DCM / MeOH solution (3 : 1) doped with 0.1 mM sodium trifluoroacetate.

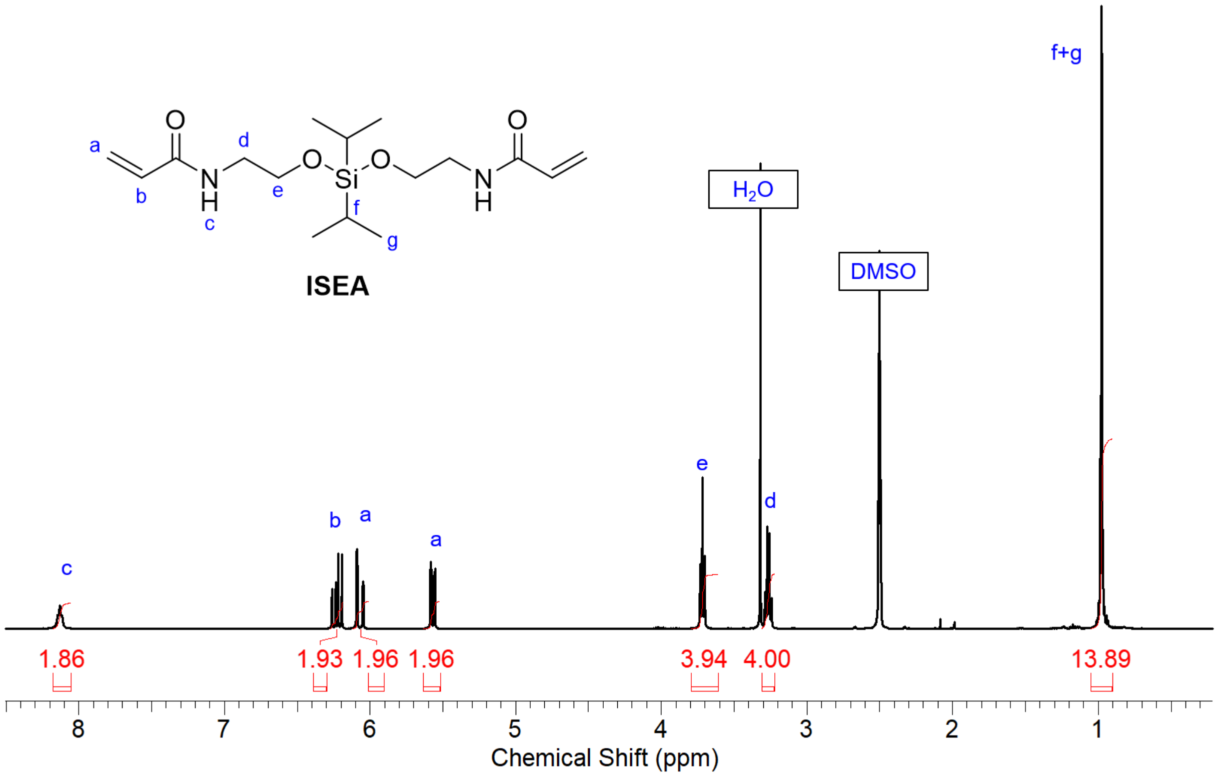

Supplementary Figure 7. ^{1}H NMR spectrum of ISEA in DMSO-d_6.

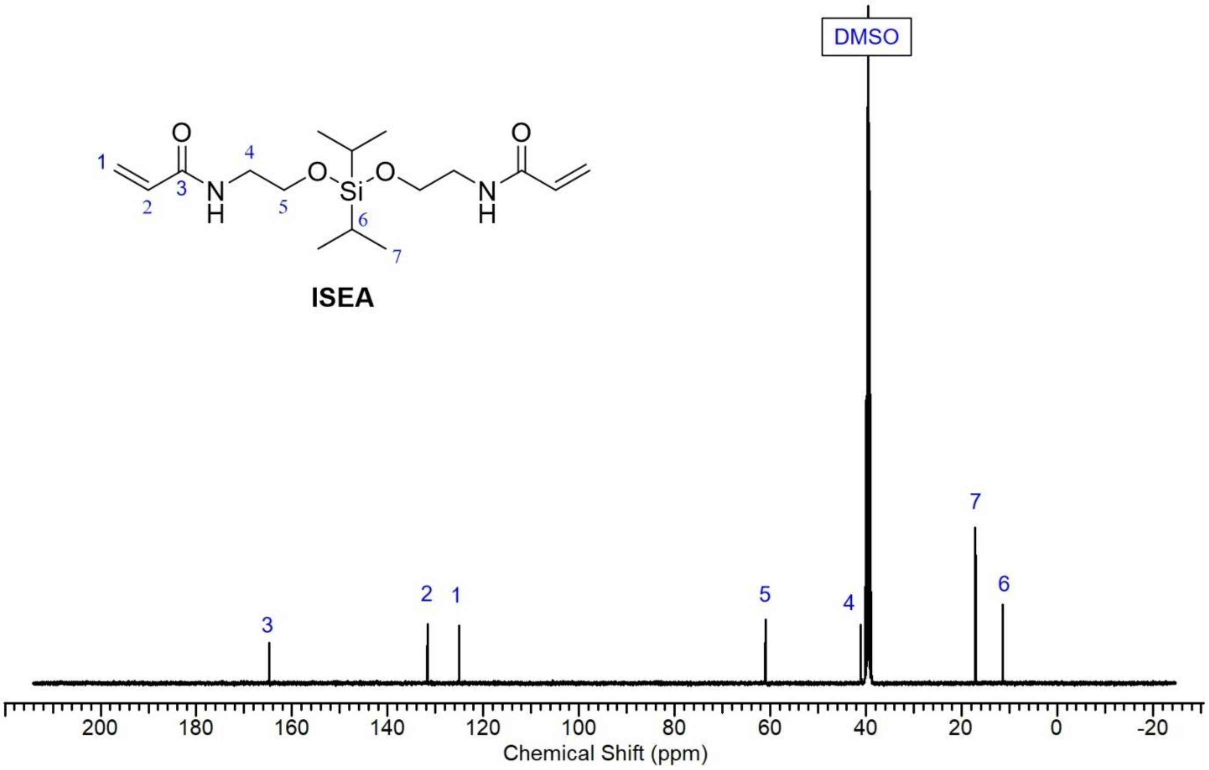

Supplementary Figure 8. ^{13}C NMR spectrum of ISEA in DMSO-d_6.

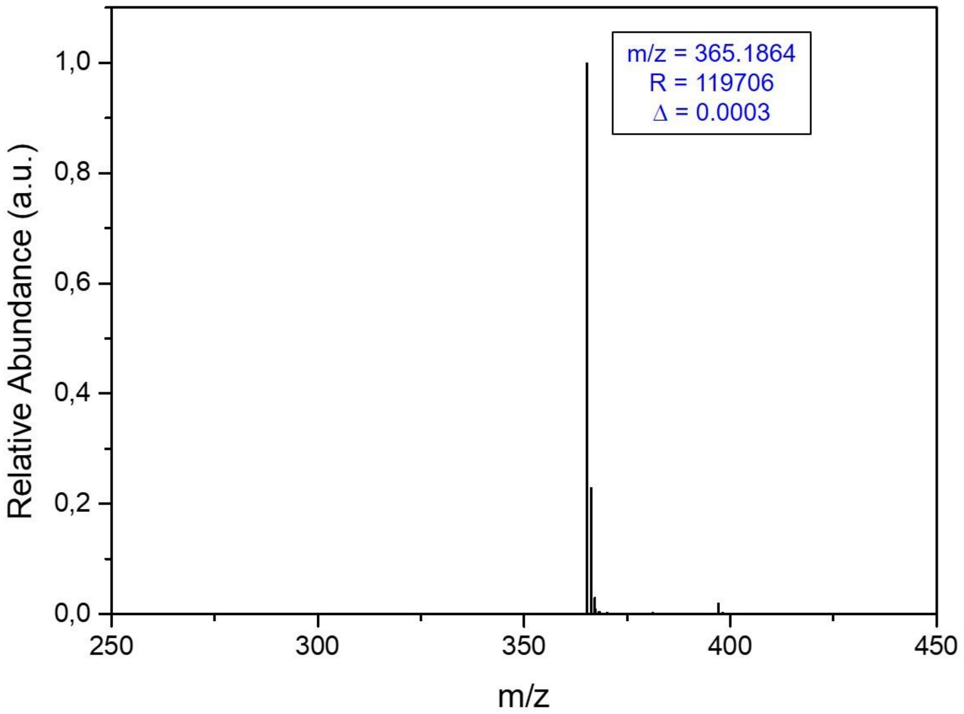

Supplementary Figure 9. ESI MS spectrum of ISEA in DCM / MeOH solution (3 : 1) doped with 0.1 mM sodium trifluoroacetate.

Supplementary Table 1. Photoresist composition of ESEA (entry 1), ISEA (entry 2), pentaerythritol triacrylate ((PETA), entry 3), MSEA+PETA (entry 4), ESEA+PETA (entry 5), ISEA+PETA (entry 6), and 95ISEA+5PETA (Entry 7).

**Entry**	**Name**	**feed ratio**		
		PETA [mmol]	Silane Crosslinker [mmol]	Irgacure 369 [mmol]
**1**	ESEA	-	250.0	1.5
**2**	ISEA	-	250.0	1.5
**3**	PETA	250.0	-	1.5
**4**	MSEA+PETA	006.2	243.8	1.5
**5**	ESEA+PETA	006.2	243.8	1.5
**6**	ISEA+PETA	006.2	243.8	1.5
**7**	95ISEA+5PETA	012.5	237.5	1.5

Supplementary Note 1

Polymer lines were fabricated to determine the minimum feature size of each photoresist mixture as shown in Supplementary Figure 10. Six different photoresist mixtures were assessed (feed ratios are shown in Supplementary Table 1). The smallest linewidth of 165 nm was measured with a photoresist composed of 100 mol% PETA. Photoresist mixtures of 97.5 mol% silane crosslinker and 2.5 mol% PETA resulted in linewidths of 170 nm and 230 nm, respectively, which are close to the reference material (*i.e.*, 100 mol% PETA). The largest linewidths were obtained for pure silane crosslinkers with values of 300 and 370 nm, respectively.

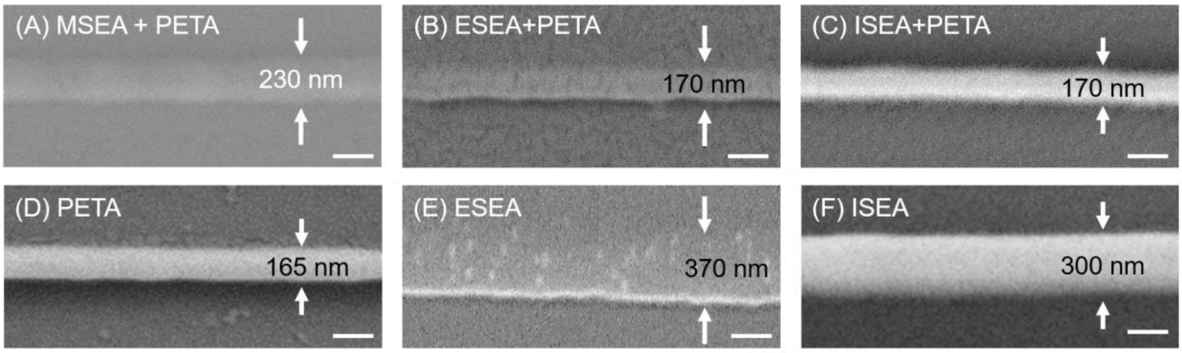

Supplementary Figure 10. SEM images of lines written on a silanized glass substrate. Feed ratios of the photoresists are shown in Supplementary Table 1. All line patterns were fabricated with a writing speed of 50 μm/s and a laser power of 5 mW. (A) Photoresist composed of 97.5 mol% MSEA mixed with 2.5 mol% PETA. (B) Photoresist composed of 97.5 mol% ESEA mixed with 2.5 mol% PETA. (C) Photoresist composed of 97.5 mol% ISEA mixed with 2.5 mol% PETA. (D) Photoresist composed of 100 mol% PETA. (E) Photoresist composed of 100 mol% ESEA. (F) Photoresist composed of 100 mol% ISEA.

Supplementary Note 2

Woodpile structures were fabricated to compare the writing properties of pure silane crosslinkers and silane crosslinkers mixed with PETA (see Supplementary Figure 11). Four different photoresist compositions were assessed (feed ratios are shown in Supplementary Table 1). Woodpiles composed of 97.5 mol% silane crosslinkers and 2.5 mol% PETA resulted in woodpiles having only the usual level of shrinkage with well separated layers and well-aligned rods without apparent defects. In contrast, woodpiles fabricated from only bifunctional silane crosslinkers were non-detailed and deformed.

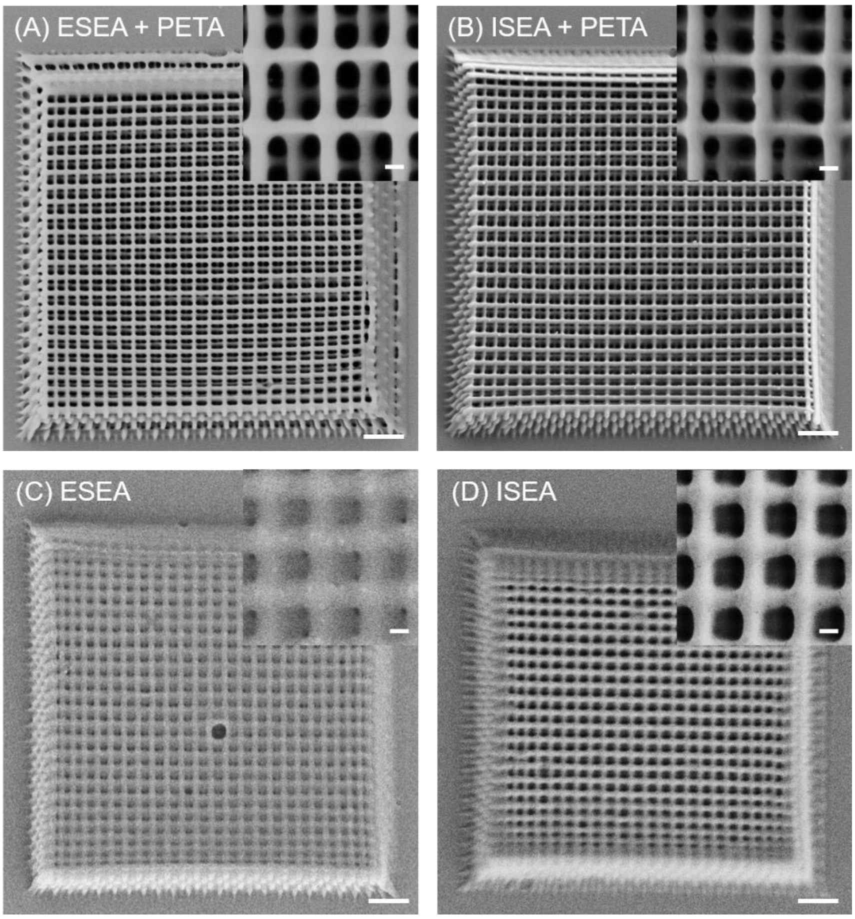

Supplementary Figure 11. SEM images of woodpile structures (12 layers, footprint of 20 μm × 20 μm, rod spacing of a = 800 nm; scale bars = 2 μm and 200 nm, respectively). All woodpiles were fabricated with a writing speed of 50 μm/s and a laser power of 5 mW. (A) Photoresist composed of 97.5 mol% ESEA and 2.5 mol% PETA. (B) Photoresist composed of 97.5 mol% ISEA and 2.5 mol% PETA. (C) Photoresist composed of 100 mol% ESEA. (D) Photoresist composed of 100 mol% ISEA.

Supplementary Figure 12 shows further 3D microstructures fabricated at various writing conditions highlighting the great writing properties of our photoresists.

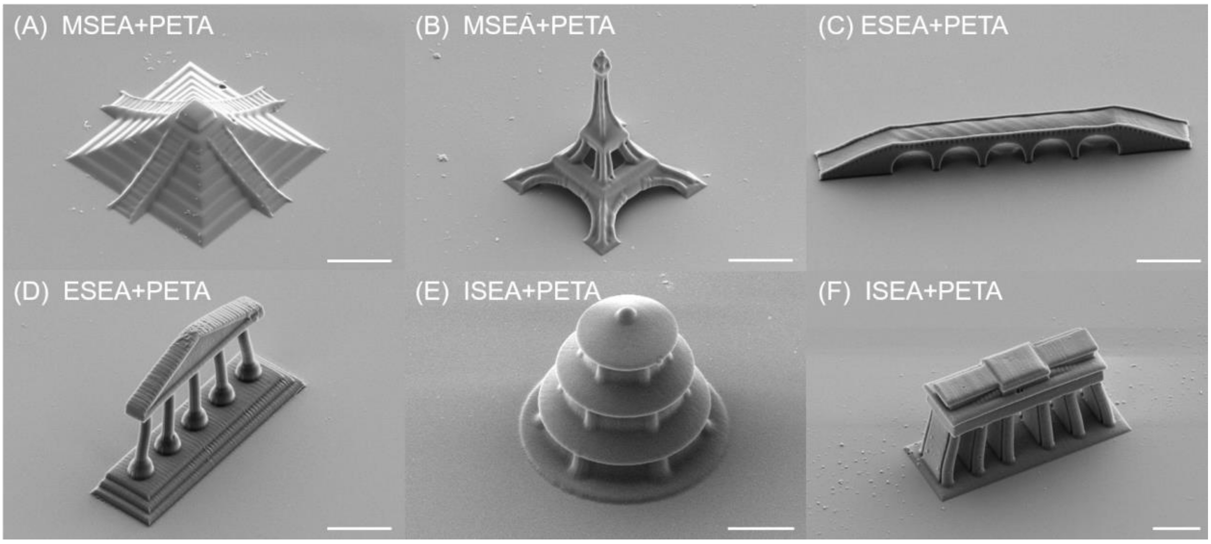

Supplementary Figure 12. SEM images of 3D structures fabricated at various writing conditions (scale bars = 10 μm). (A) Aztec pyramid composed of 97.5 mol% MSEA and 2.5 mol% PETA (writing speed = 1 mm/s; laser power = 12.5 mW). (B) Eiffel tower composed of 97.5 mol% MSEA and 2.5 mol% PETA (writing speed = 3 mm/s; laser power = 21 mW). (C) Bridge composed of 97.5 mol% ESEA and 2.5 mol% PETA (writing speed = 1 mm/s; laser power = 15 mW). (D) Greek temple composed of 97.5 mol% ESEA and 2.5 mol% PETA (writing speed = 1 mm; laser power = 12.5 mW). (E) Asian temple composed of 97.5 mol% ISEA and 2.5 mol% PETA (writing speed = 10 mm; laser power = 21.5 mW). (F) Brandenburg Gate composed of 97.5 mol% ISEA and 2.5 mol% PETA (writing speed = 10 mm; laser power = 21.5 mW)

Supplementary Note 3

To determine the percentage of shrinkage (PS), we used woodpile structures as presented in Supplementary Figure 13.

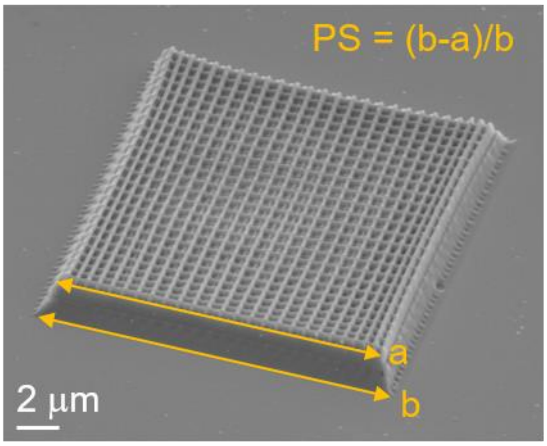

Supplementary Figure 13. SEM image of a woodpile structure to determine the percentage of shrinkage (PS).

The PS was determined by measuring the length difference between the top and bottom layers of the woodpile structure. Supplementary Table 2 summarizes Percentage of shrinkage (PS), length of the top layer (a) and bottom layer (b) for PETA, MSEA, ESEA and ISEA.

Supplementary Table 2. Percentage of shrinkage (PS), length of the top layer (a) and bottom layer (b) for PETA, MSEA, ESEA and ISEA.

photoresist	a [μm]	b [μm]	PS [%]
PETA	18.8	20	6
MSEA	17.7	20	12
ESEA	17.3	20	14
ISEA	18.1	20	10

Supplementary Note 4

We investigated the effect of PETA fraction within the resist on the degradation properties of the resulting 3D microstructures. Three photoresist mixtures were assessed (feed ratios are shown in Supplementary Table 1): (1) 100 mol% PETA (Entry 4), (2) 97.5 mol% ISEA and 2.5 mol% PETA (Entry 6), and 95 mol% ISEA and 5 mol% PETA (Entry 7). Using these photoresist mixtures, polymer blocks were fabricated and subsequently immersed in a saturated solution of KF in MeOH. Supplementary Figure 14 shows a representative SEM image of polymer blocks used for the degradation studies.

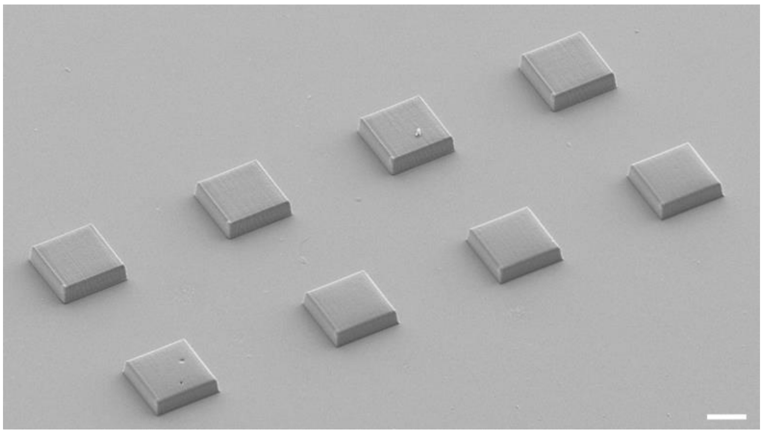

Supplementary Figure 14. SEM image of polymer blocks used for the cleavage studies (scale bar = 10 μm). The dimension of the blocks is 20 x 20 x 5 μm^3 (L x W x H). Polymer blocks were fabricated at a writing speed of 0.5 mm/s and a laser power of 12.5 mW.

As presented in Supplementary Figure 15, optical microscope images revealed that polymer blocks made of PETA remain entirely unaffected. Polymer blocks composed of 97.5 mol% ISEA and 2.5 mol% PETA disappeared completely after 1 h. In contrast, polymer blocks made of 95 mol% ISEA and 5 mol% PETA degraded just partially within 4 h. The polymer blocks composed of 95 mol% ISEA and 5 mol% PETA are still recognizable in the optical microscope images. As rapid and complete cleavage is necessary for an industrial relevant application, PETA amount was kept at 2.5 mol%.

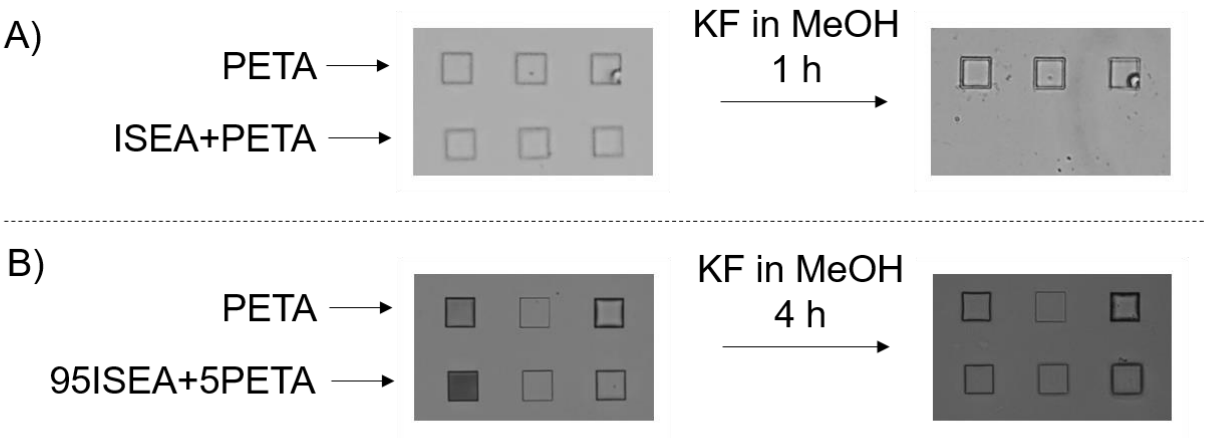

Supplementary Figure 15. Optical microscope images of polymer blocks made of different photoresists before and after immersing in a saturated solution of KF in MeOH. Feed ratios are shown in Supplementary Table 1. (A) Two rows of polymer blocks composed of either 100 mol% PETA (upper row, Entry 4 in Supplementary Table 1) or 97.5 mol% ISEA and 2.5 mol% PETA (lower row, Entry 6). (B) Two rows of polymer blocks composed of either 100 mol% PETA (upper row, Entry 4) or 95 mol% ISEA and 5 mol% PETA (lower row, Entry 7).

Supplementary Note 4

Silane crosslinker MSEA. We investigated the degradation of 3D microstructures composed of either 100 mol% PETA (Entry 3 in Supplementary Table 1) or 97.5 mol% MSEA and 2.5 mol% PETA (Entry 4) in a saturated solution of $NaHCO_3$ in MeOH at 50 °C. Supplementary Figure 16 shows a representative SEM image of the model structure (*i.e.*, Eiffel tower) used in the degradation experiments. Time-lapse optical microscope images revealed that the Eiffel tower fabricated from 97.5 mol% MSEA and 2.5 mol% PETA entirely degraded after 20 minutes. In contrast, the reference structure composed of 100 mol% PETA remained unaffected.

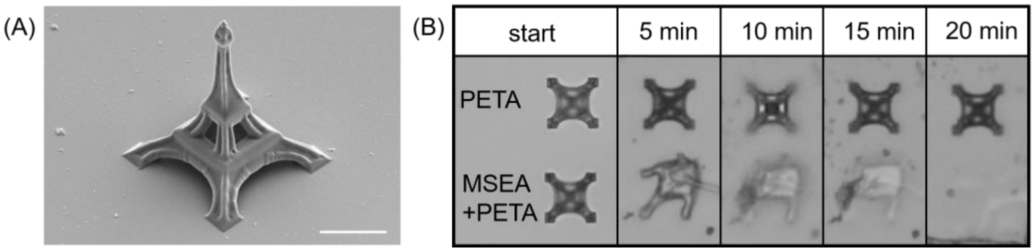

Supplementary Figure 16. (A) Representative SEM image of an Eiffel tower used in the degradation experiment (scale bar = 10 μm). The model structures were fabricated at a writing speed of 3 mm/s and a laser power of 21 mW. (B) Time-lapse optical microscope images of two Eiffel towers immersed in a saturated solution of $NaHCO_3$ in MeOH at 50 °C. The Eiffel towers were fabricated either with 100 mol% PETA or 97.5 mol% MSEA and 2.5 mol% PETA.

Silane crosslinker ESEA. We investigated the degradation of 3D microstructures composed of either 100 mol% PETA (Entry 3 in Supplementary Table 1) or 97.5 mol% ESEA and 2.5 mol% PETA (Entry 5) in a saturated solution of K_2CO_3 in MeOH at room temperature (RT). Supplementary Figure 17 shows a representative SEM image of the model structure (*i.e.*, Aztec pyramid) used in the degradation experiments. Time-lapse optical microscope images revealed that the Aztec pyramid made of 97.5 mol% ESEA and 2.5 mol% PETA entirely degraded after 50 minutes. In contrast, the reference structure composed of 100 mol% PETA remained unaffected.

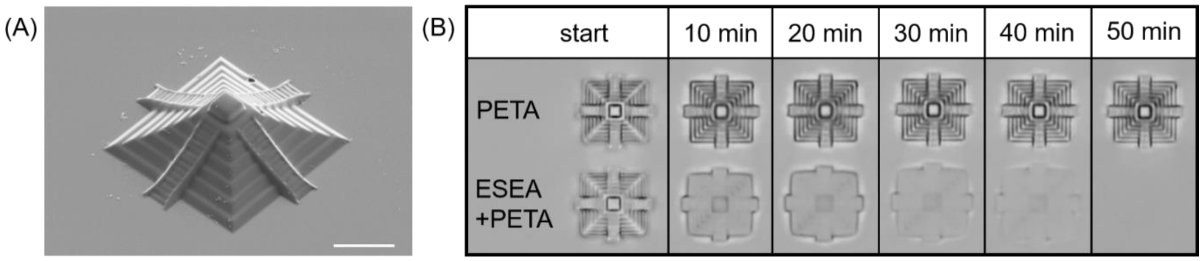

Supplementary Figure 17. (A) Representative SEM image of an Aztec pyramid used in the degradation experiment (scale bar = 10 μm). The model structures were fabricated at a writing speed of 1 mm/s and a laser power of 12.5 mW. (B) Time-lapse optical microscope images of two Aztec pyramids immersed in a saturated solution of K_2CO_3 in MeOH at RT. The Aztec pyramids were fabricated either with 100 mol% PETA or 97.5 mol% ESEA and 2.5 mol% PETA.

Silane crosslinker ISEA. We investigated the degradation of 3D microstructures composed of either 100 mol% PETA (Entry 3 in Supplementary Table 1) or 97.5 mol% ISEA and 2.5 mol% PETA (Entry 6) in a saturated solution of KF in MeOH at RT. Supplementary Figure 18 shows a representative SEM image of the model structure (*i.e.*, a bridge) used in the degradation experiments. Time-lapse optical microscope images revealed that the bridge made of 97.5 mol% ISEA and 2.5 mol% PETA entirely degraded after 60 minutes. In contrast, the reference structure composed of 100 mol% PETA remained unaffected.

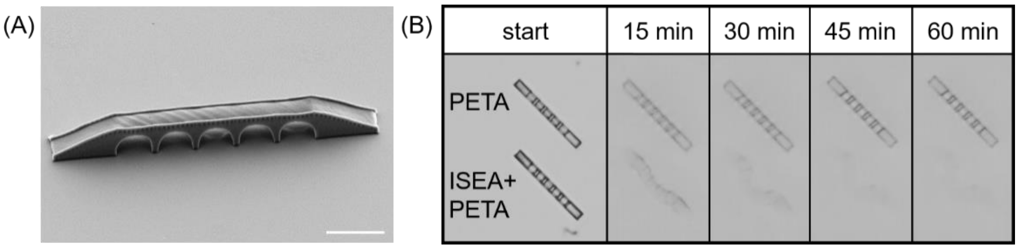

Supplementary Figure 18. (A) Representative SEM image of a bridge used in the degradation experiment (scale bar = 10 μm). The model structures were fabricated at a writing speed of 1 mm/s and a laser power of 15 mW. (B) Time-lapse optical microscope images of two bridges immersed in a saturated solution of KF in MeOH at RT. The bridge were fabricated either with 100 mol% PETA or 97.5 mol% ISEA and 2.5 mol% PETA.

Supplementary Note 5

We fabricated glass substrates containing model structures made of four different photoresists: (1) 97.5 mol% MSEA and 2.5 mol% PETA (Entry 4 in Supplementary Table 1), (2) 97.5 mol% ESEA and 2.5 mol% PETA (Entry 5), (3) 97.5 mol% ISEA and 2.5 mol% PETA (Entry 6) and (4) 100 mol% PETA (Asian temple, Entry 3). Supplementary Figure 19 shows representative images of the used glass substrate.

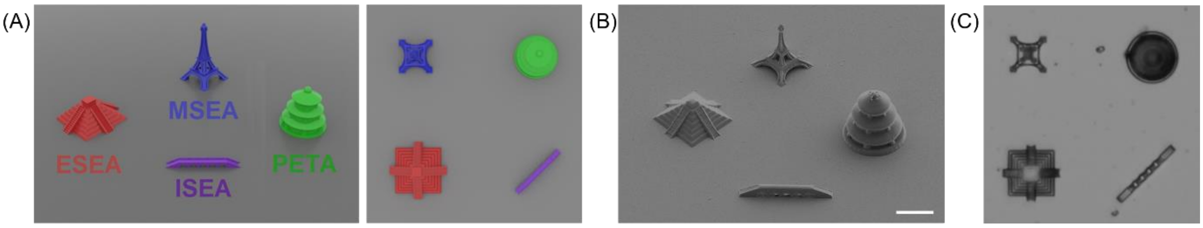

Supplementary Figure 19. (A) Schematic representation of a glass substrate containing model structures made of four different photoresists. The Eiffel tower (blue structure, laser power = 21 mW, writing speed = 3 mm/s) was prepared using MSEA, the Aztec pyramid (red structure, laser power = 12.5 mW, writing speed = 1 mm/s) was made of ESEA, the bridge (purple structure, laser power =12.5 mW, writing speed = 1 mm/s) was prepared using ISEA and the Asian temple (green structure, laser power = 12.5 mW, writing speed = 1 mm/s) was fabricated from PETA. The colour code of the left image (front view) corresponds to the colour code of the right image (top view). (B-C) Representative SEM (B) and optical microscope (C) images of a glass substrate containing model structures made of four different photoresists.

We subjected the glass substrates to three conditions: (1) a saturated solution of $NaHCO_3$ in MeOH at 50 °C, (2) a saturated solution of K_2CO_3 in MeOH at room temperature (RT), and (3) a saturated solution of KF in MeOH at RT. Time-lapse images using SEM and optical microscopy are shown in Supplementary Figures 20.

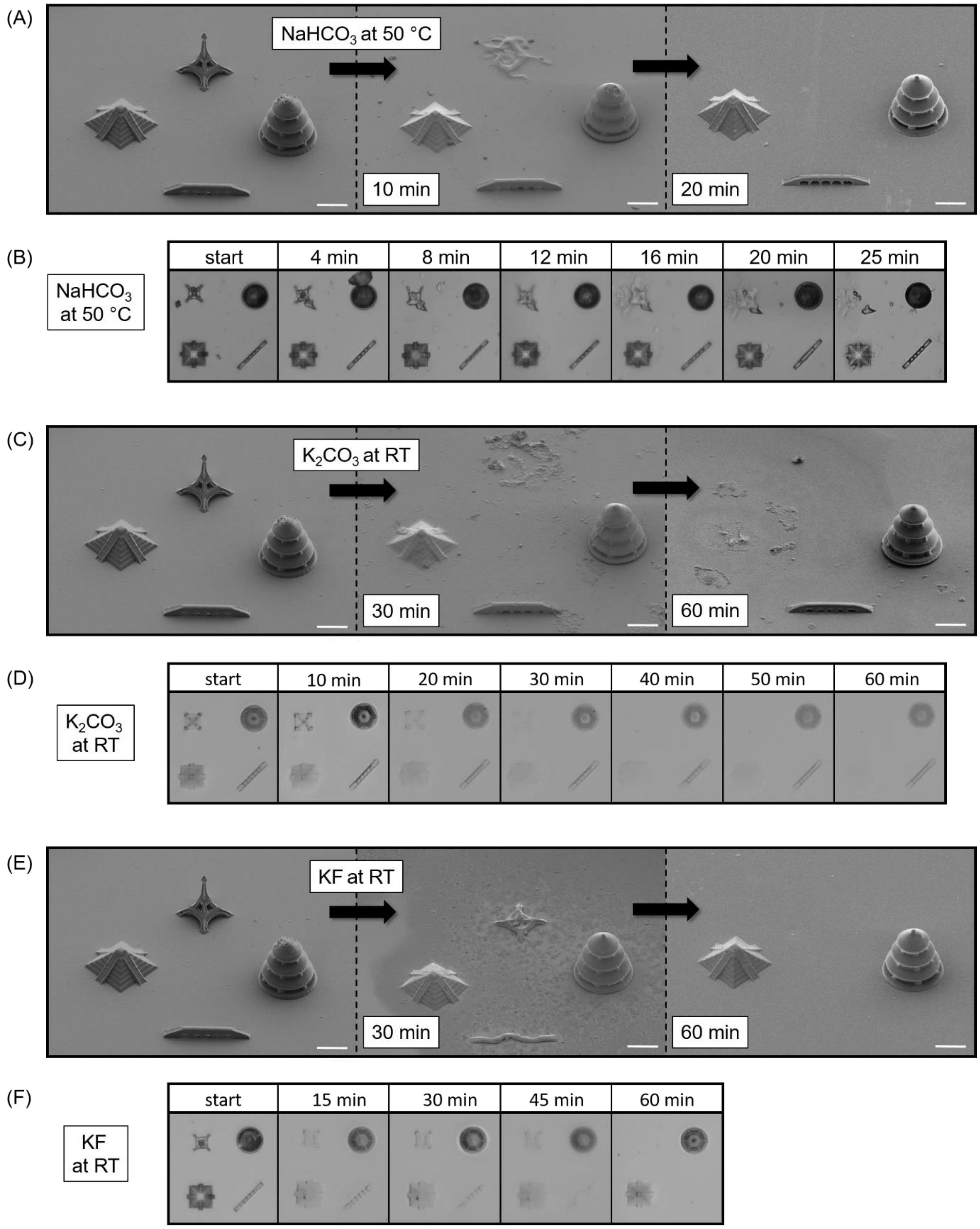

Supplementary Figure 20. (A) SEM and (B) Time-lapse optical microscope images of a substrate containing model structures made of four different photoresists immersed in a saturated solution of $NaHCO_3$ in MeOH at 50 °C. (C) SEM and (D) Time-lapse optical microscope images of a substrate containing model structures made of four different photoresists immersed in a saturated solution of K_2CO_3 in MeOH at RT. (E) SEM and (F) Time-lapse optical microscope images of a substrate containing model structures made of four

different photoresists immersed in a saturated solution of KF in MeOH at RT. Scale bars = 20 μm.

Supplementary Note 6

We fabricated glass substrates containing model structures made of four different photoresist as shown in Supplementary Figure 21. The glass substrate was sequentially immersed in $NaHCO_3$ at 50 °C, then, in K_2CO_3 at RT and finally, in KF at RT. Supplementary Figure 21 shows time-lapse optical microscope images of each individual cleavage step.

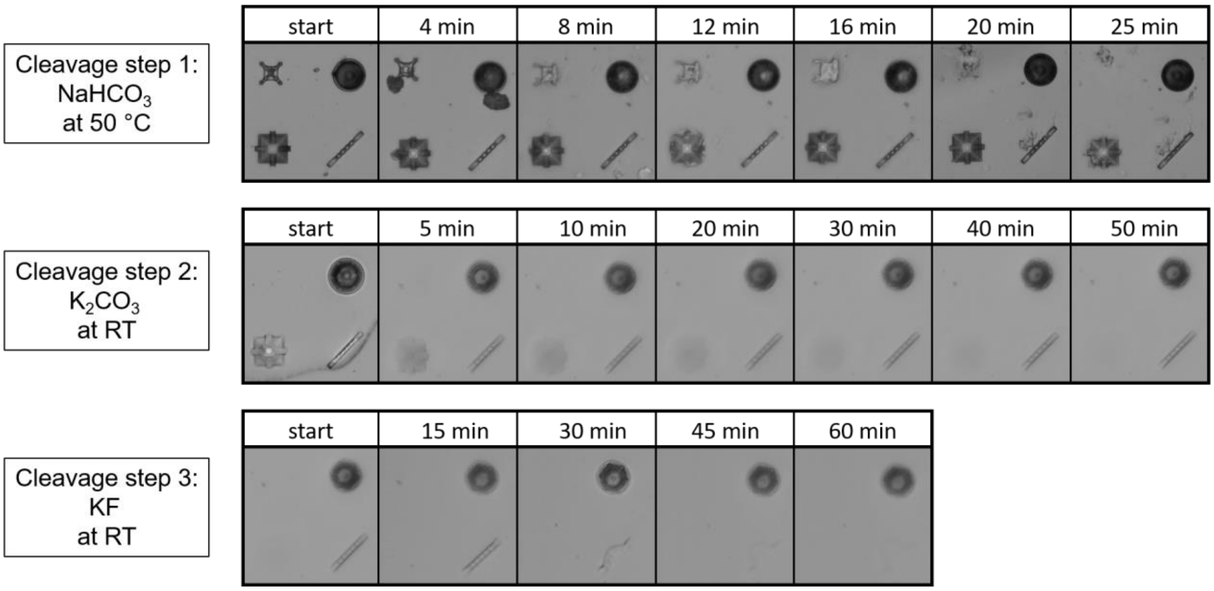

Supplementary Figure 21. Time-lapse optical microscope images of a glass substrate subjected sequentially to three conditions: first, a saturated solution of $NaHCO_3$ in MeOH at 50 °C, then, a saturated solution of K_2CO_3 in MeOH at RT, and finally, a saturated solution of KF in MeOH at RT.
